# Supplementary material for: Investigating the Physiological and Molecular Responses of Solanum lycopersicum hp Mutants to Light of Different Quality for Biotechnological Applications
Source: Int J Mol Sci. 2023 Jun 15;24(12):10149. doi: 10.3390/ijms241210149 (PMC10299061; doi:10.3390/ijms241210149)
Supplement: Supplementary file 1 [file ijms-24-10149-s001.zip › ijms-2438330-supplementary.pdf]

Table S1. Table of primers, as well as explanations of the functions of the genes studied in the research.

	Gene Bank ID	Gene description	Gene	Primer 5′-3′	
				Forward	Reverse
1	NM_001320396.1	SPA nuclear gene for chloroplast product	*SPA*	AGAACACTCAGCCCTGCTTC	GGTACCGAGGTTGAATGCCA
2	EU218537.1	Cullin 4	*CUL*	AGCCAGAACAAAGCTCCCAA	ACGCCTACCACTGTACTTGC
3	XM_004248193.4	Light-dependent short hypocotyls 10-like	*HY10*	TCAACGACCGCCTATATCGTT	TAGGCTTCCCCATGCTTGTC
4	Solyc09g075080.3.1	Phytochrome a-associated f-box protein	*PHYA-F*	CCGTGAGCAGGGAAATAAGC	GATCGATCTTCCTATTGCCATCA
5	NM_00124747191.2	Short hypocotyls 5	*HY5*	ACCCGACCCAAACATTGTCG	GGCTAGGGTTAATGGCGGTT
6	NM_001247104.2	Chalcone synthase	*CHS*	CCGTGGACCCAGTGAATCTC	AGAGTTTGGGCTGCTGAGAC
7	XM_004249510.4	Phenylalanine ammonia-lyase 1	*PAL1*	GAAGCGTTCCATGTTGCTGG	TCAGTGAACTCGGGCTTTCC
8	NM_001247883.2	Phytoene synthase	*PSY*	CGGGGAATTTGGGCTTGTTG	CCACCTATCTAAGGCTGCCG
9	NM_001308008.1	Phytochrome-interacting factor 4	*PIF4*	AATCTGGAACTGCCCGAAGG	GCCACTTCCCATCCACATCA
10	A0A3Q7F8W6	Tubulin alpha chain	*TUB*	ACAACTTTGCCCGTGGACAT	TGCTCAAGAAGGGAGTGGGT
11	NM_001247118.2	E3 ubiquitin-protein ligase	*COP1*	ATAATTTGCCGGTTCGCACG	CACGGGTTTGGATATCGCCT
12	NM_001247219.2	De-etiolated1	*DET1*	TGATGGCTTCCTTGCCTTGT	CCATCTGTGCTGCCAGCTTC

SPA nuclear gene for chloroplast product (Suppressor of Phytochrome A) proteins are key components of the light-signaling pathway in plants. They function as negative regulators of light signaling, particularly in the dark or in low-light conditions. SPA proteins form a complex with the E3 ubiquitin ligase COP1 (Constitutive Photomorphogenic 1), and this COP1/SPA complex targets certain transcription factors for degradation, thereby suppressing photomorphogenic development.

Cullin 4 is a core component of a class of E3 ubiquitin ligases. In plants, these multiprotein complexes are involved in the ubiquitination of specific target proteins, marking them for degradation by the 26S proteasome. This is a fundamental process for modulating protein levels in cells, thereby controlling various cellular processes.CUL4, specifically, has been implicated in the regulation of photomorphogenesis in plants, which is the process of growth and development in response to light. It forms a complex with DDB1 (Damaged DNA Binding protein 1) and DET1 (De-Etiolated 1). This CUL4-DDB1-DET1 complex is involved in the degradation of positive regulators of light signaling, thus acting as a negative regulator of photomorphogenesis in darkness. When plants are exposed to light, this degradation activity is suppressed, enabling photomorphogenesis to occur. In this way, CUL4 plays a key role in controlling plant responses to light.

Light-dependent short hypocotyls 10-like is a transcriptional factor part of the light signaling pathway in plants, particularly involved in the photomorphogenesis process, i.e., the growth and development of a plant in response to light.

Phytochrome a-associated f-box protein Phytochrome A-associated F-box protein (PHYA-F) is believed to interact with PHYA as part of the light signaling pathway. F-box proteins are part of the SCF (SKP1-CUL1-F-box protein) E3 ubiquitin ligase complex, which is responsible for targeting specific proteins for ubiquitination and subsequent degradation by the 26S proteasome.

Short hypocotyls 5 HY5 promotes photomorphogenesis in response to light signals. This promotes the development of photosynthetic tissues and inhibits elongation growth, leading to characteristics typical of light-grown plants, such as short hypocotyls and open cotyledons. In darkness, HY5 is targeted for degradation by the COP1/SPA E3 ubiquitin ligase complex, thus inhibiting photomorphogenesis. However, in the presence of light, COP1/SPA activity is suppressed, allowing HY5 to accumulate and promote photomorphogenesis.

Chalcone synthase The primary role of CHS is to catalyze the first committed step in the flavonoid biosynthetic pathway, leading to the production of a variety of flavonoid compounds. This step involves the condensation of one molecule of 4-coumaroyl-CoA with three molecules of malonyl-CoA to produce chalcone. Chalcone synthase (CHS) is a key enzyme involved in the biosynthesis of flavonoids in plants. Flavonoids are a diverse group of secondary metabolites that perform a variety of functions.

Phenylalanine ammonia-lyase 1 PAL1 is an important enzyme in plants that plays a critical role in the biosynthesis of phenylpropanoids, a large class of secondary metabolites.The role of PAL1 is to catalyze the deamination of the amino acid phenylalanine to trans-cinnamic acid, which is the first and typically rate-limiting step in the phenylpropanoid pathway.

Phytoene synthase PSY is an enzyme that plays a key role in carotenoid biosynthesis in plants. Carotenoids are a class of pigments that are involved in various functions in plants, including photosynthesis and photoprotection.The specific role of PSY is to catalyze the first committed step in the carotenoid biosynthetic pathway.

Phytochrome-interacting factor 4 PIF4 is a key regulatory protein involved in the phytochrome signaling pathway in plants. This pathway is responsible for mediating various responses to light, particularly red and far-red light.PIFs are a group of transcription factors that bind to phytochromes, the light-absorbing proteins that initiate the light response pathway. When light is absorbed, phytochromes undergo a conformational change and translocate to the nucleus where they interact with PIF4.

E3 CCU-protein ligase COP1 Constitutive Photomorphogenic 1 is a key protein in plants that plays an important role in light signal transduction pathways. COP1 is particularly significant in photomorphogenesis, the growth and development of plants in response to light. In darkness, COP1 is active and localizes to the nucleus where it targets various light-responsive transcription factors for degradation through the ubiquitin-proteasome pathway. Among these targets

are proteins like ELONGATED HYPOCOTYL 5 (HY5) and other members of the bZIP transcription factor family, which promote photomorphogenic development. By targeting these proteins for degradation, COP1 suppresses photomorphogenesis, promoting skotomorphogenesis (growth in darkness) characterized by features such as elongated hypocotyls and closed cotyledons.

De-etiolated1 DET1 is a key regulator protein involved in controlling photomorphogenesis in plants. Photomorphogenesis is the process by which plants alter their growth and development in response to light.In darkness, DET1 acts to suppress photomorphogenesis and promote skotomorphogenesis, which is growth in darkness characterized by features such as elongated hypocotyls, closed cotyledons, and underdeveloped chloroplasts. DET1 is part of a multi-protein complex that targets positive regulators of light signaling, including transcription factors like HY5, for degradation via the ubiquitin-proteasome pathway.
